# Echocardiographic Assessment of Pulmonary Hemodynamics and Right Ventricular Performance in Neonatal Murine Hypoxia

**DOI:** 10.3390/jcdd12080316

**Published:** 2025-08-19

**Authors:** Kel Vin Woo, Philip T. Levy, Carla J. Weinheimer, Amanda L. Hauck, Aaron Hamvas, David M. Ornitz, Attila Kovacs, Gautam K. Singh

**Affiliations:** 1 Department of Pediatrics, Division of Cardiology, Washington University School of Medicine, Saint Louis, MO 63110, USA; 2 Department of Developmental Biology, Washington University School of Medicine, Saint Louis, MO 63110, USA; dornitz@wustl.edu; 3 Division of Newborn Medicine, Boston Children’s Hospital, Department of Pediatrics, Harvard Medical School, Boston, MA 02115, USA; philip.levy@childrens.harvard.edu; 4 Cardiovascular Division, Department of Medicine, Washington University School of Medicine, Saint Louis, MO 63110, USA; carlajweinheimer@wustl.edu (C.J.W.); akovacs@wustl.edu (A.K.); 5 Department of Pediatrics, Ann and Robert H. Lurie Children’s Hospital of Chicago, Northwestern University Feinberg School of Medicine, Chicago, IL 60611, USA; ahauck@luriechildrens.org (A.L.H.); ahamvas@luriechildrens.org (A.H.); 6 Division of Pediatric Cardiology, Children’s Hospital Michigan and Central Michigan University, Detroit, MI 48201, USA; gsingh3@dmc.org

**Keywords:** prematurity, echocardiography, pulmonary hypertension, animal models, right ventricle

## Abstract

**Background:** Right heart catheterization (RHC) is the gold-standard for diagnosis of pulmonary hypertension (PH) but is a terminal procedure in neonatal mice. The objective was to validate echocardiographic measures of PH to establish the diagnostic capability against pulmonary vascular histology in neonatal mice. **Methods:** Adult mice, exposed to hypoxia or normoxia, were assessed by echocardiography and RHC to evaluate right ventricle (RV) morphometry and function. Echocardiographic measures identified in adult mice were then used to evaluate PH characteristics in hypoxia-exposed neonatal mice. Physiological parameters were compared to histopathology in all mice. **Results:** Hypoxia-challenged adult mice developed PH with RHC, demonstrating confirmed elevated RV systolic pressure (RVSP), RV hypertrophy, and increased cross-sectional area and neomuscularization of pulmonary vessels. Echocardiography-derived RV free wall (RVFW) thickness correlated with RV mass. Tricuspid valve annulus tissue Doppler imaging (TV TDI), tricuspid annular plane systolic excursion (TAPSE), pulmonary artery acceleration measures (PAAT), and TAPSE × PAAT (a measure of RV work) all correlated with RVSP determined by RHC. In neonatal mice exposed to hypoxia, PAAT, TV TDI, TAPSE, and TAPSE × PAAT were decreased and RVFW thickness was increased, correlating with the histologic phenotype of PH. **Conclusions:** Echocardiographic indices of RV morphology and function provide reliable estimates of invasive RV hemodynamics in hypoxia-induced PH.

## 1. Introduction

Pulmonary vascular disease (PVD) and its most severe form, pulmonary hypertension (PH), are associated with neonatal disorders of hypoxia, including bronchopulmonary dysplasia (BPD) [1]. Hypoxia-induced PH is characterized by progressive obliterative vasculopathy, elevated right ventricular (RV) afterload and high mortality from right heart failure. Right heart catheterization (RHC) is the gold standard for the establishment of the diagnosis, evaluation of RV-pulmonary artery (PA) coupling, and for drug testing to guide treatment. However, the invasive nature and risk of radiation exposure limits the use of RHC as a diagnostic tool in premature infants and as a screening and monitoring tool in pediatric patients [2]. Up to 40% of premature infants with BPD develop hypoxia-induced PH (World health organization (WHO) classification Group 3 PH), which is associated with up to 50% mortality by two years of age [1,3]. Despite the increased recognition of hypoxia induced PH in preterm infants with BPD, the understanding of PH in premature neonates remains poor due to a lack of reliable models to study pulmonary hemodynamics and RV performance [1,4,5].

Hypoxia challenged neonatal mice develop pulmonary vascular remodeling [6] that mimics PVD seen in premature born infants with BPD [7,8]. RV systolic pressure (RVSP) measured by RHC is usually accepted as a surrogate to pulmonary artery systolic pressure (PASP) and serves as the principal diagnostic marker of PH in adult mice. However, RHC is a terminal procedure in mice that does not allow longitudinal monitoring [9]. This limitation is further amplified because RHC cannot technically be performed in neonatal mice at 2 weeks of age, the appropriate age for disease modeling due to prematurity, which is developmentally similar to that of human infants born at 28 weeks’ gestation. Studies in neonatal mice currently rely on histology of postmortem heart and lung tissue to formally diagnose PH, impeding serial evaluation [9].

In neonatal mice, 2D echocardiography offers a practical, noninvasive method for diagnosing and monitoring RV hemodynamics, structure, and function. Several measures of RV mechanics, derived from adult mouse studies with pulmonary hypertension, are available [9,10,11,12]. These include evaluation of (1) RV morphology, such as RV free wall thickness; (2) RV function, using tissue Doppler imaging (TDI) for RV myocardial velocities and tricuspid annular plane systolic excursion (TAPSE); and (3) RV afterload, measures through pulmonary artery acceleration time (PAAT) [2]. Although echocardiographic measures are considered a reliable surrogate for RHC measures and allow for serial investigations in adult mice, they have not been characterized for PH in neonatal mice. Furthermore, the relationship of recently described novel echocardiographic hemodynamic parameters that describe the interactions between the RV and its afterload (coupling) [13,14] to physiologic and histologic changes in adult and neonatal mice with PH have never been investigated in a murine model. Understanding the correlation of all these echocardiographic parameters to physiological and histologic changes in neonatal mice may offer insights into the disease mechanisms in premature infants.

We propose that noninvasive echocardiographic indicators of RV function in neonatal mice could provide reliable estimates of RV systolic pressure (RVSP), as measured by right heart catheterization. To test this, we used a well-known PH mouse model involving two weeks of chronic hypoxia exposure (10% oxygen) [11]. In the initial phase (derivation), we performed simultaneous echocardiography and right heart catheterization on adult mice. These echocardiographic measures were then used to assess PH in neonatal mice during the validation phase of the study.

## 2. Materials and Methods

### 2.1. Study Design

This study consisted of two parts, a derivation and a validation component. The derivation part consisted of a study of 6–8 weeks-old adult mice housed in either normoxia (males *n* = 5, females *n* = 2) or hypoxia (males *n* = 4, females *n* = 2) for 2 weeks. Chronic hypoxia commonly causes medial thickening and muscularization of vessels in mice, leading to elevated pulmonary artery pressure [9]. The adult mice underwent echocardiography and cardiac catheterization at the end of the 2 weeks to assess the validity of echocardiographic measures of RV function and loads in comparison to RHC. The experimental part was related to neonatal-young infant equivalent mice who were exposed to either normoxia (males *n* = 3, females *n* = 3) or hypoxia (males *n* = 7, females *n* = 3) from birth to 2 weeks of age. The neonatal mice only underwent echocardiography at the conclusion of the two weeks to evaluate the feasibility and reliability of the echocardiographic measures that were observed in the adult mice. The Washington University Animal Care and Use Committee approved all animal experiments.

### 2.2. Animal Care and Use

Mice were housed in a specific pathogen-free barrier facility and handled in accordance with standard protocols and animal welfare regulations. All procedures complied with the Guide for the Care and Use of Laboratory Animals (NIH publication No 85–23, revised 1996), and all protocols were approved by the Animal Studies Committee at Washington University School of Medicine.

### 2.3. Animal Model of Hypoxia-Induced PH

To mitigate a potential genetic background effect, all experimental mice were maintained on a mixed C57BL/6J and 129X1/SvJ genetic background. Adult male and female mice at 6–8 weeks of age were divided into two experimental groups based on exposure to normoxia and hypoxia. Hypoxic groups were kept in a ventilated hypoxic cabinet (Coy Laboratory Products, Grass Lake, MI, USA) for 14 days with fraction of oxygen in the inspired air (FiO_2_) at 10%. Normoxic groups were kept in room air for 14 days. Routine husbandry was performed within the chamber with no interruption of oxygen levels. At the end of the hypoxia exposure period, the adult hypoxia and normoxia groups were analyzed by echocardiography performed by a blinded sonographer. The adult hypoxic mice were placed back in the hypoxia chamber for at least 2 h after echocardiography. Both groups were then analyzed by RHC. Histology was performed in both adult mice groups.

Neonatal pups on postnatal day 0 with nursing dams were placed in the hypoxic cabinet or kept in room air for 14 days. Routine husbandry was performed within the chamber with no interruption of oxygen levels. At the end of the hypoxia exposure period, the neonatal hypoxia and normoxia mice were analyzed by echocardiography performed by a blinded sonographer. Histology was performed in both groups. There was no mortality in either the adult or neonatal groups associated with hypoxia exposure.

### 2.4. Echocardiography

Transthoracic 2D M-mode and Doppler imaging were performed in the Washington University Mouse Cardiovascular Phenotyping Core facility (https://mcpc.wustl.edu/, accessed on 30 May 2025) using a VisualSonics Vevo 2100 In Vivo Imaging System (Visual Sonics, Toronto, ON, Canada) according to the guidelines of the American Society of Echocardiography. Mice were anesthetized with Avertin (2,2,2-tribromoethanol, 100 mg/kg, i.p.). Avertin was chosen due to its lack of cardio-depressive effects at the doses administered in this study.

To analyze RV longitudinal function, images were acquired from the apical 4-chamber view. A M-mode cursor was placed through the lateral annulus of the tricuspid valve (TV) plane to measure tricuspid annular plane systolic excursion (TAPSE) [15], defined as the distance of excursion of the TV annulus from the base towards the apex during systole [16]. TAPSE has been shown to correlate well with RV ejection fraction measured by MRI [12]. Tissue Doppler imaging myocardial velocities were obtained in the apical 4-chamber view at the TV lateral annulus as systolic velocity (s’) and early diastolic velocity (e’) [17]. RV morphology was assessed with end-diastolic RV free wall thickness (RVFW) in a modified right parasternal long-axis view using a M-mode sample volume across the RV wall perpendicular to the RV long axis at the level of the mitral valve annulus [18]. For all parameters a minimum of 5 values were measured from each recording and averaged.

Pulmonary hemodynamics were assessed through images acquired from the left parasternal short-axis view. Color pulsed Doppler was used with sample volume placed in the center of the color Doppler of main PA and interrogated along the line of flow. To interrogate pulmonary artery flow and RV outflow tract systolic time intervals, PAAT was measured from the interval between the onset of systolic ejection and the peak flow velocity. RV ejection time (RVET) was also measured from the interval between the onset of RV ejection to the point of systolic pulmonary arterial flow cessation [2]. To account for heart rate variability, PAAT was adjusted to RVET and was presented as PAATi (PAAT/RVET). PAAT and PAATi are non-invasive measures of RV afterload that provide an accurate estimate of invasive PVR, PA pressure, and PA compliance in children with PH [2] and in hypoxia PH mouse models [19].

Since optimal RV performance in PH requires structural and functional coupling between the RV and its adjacent pulmonary vascular network [20], recent studies in humans have also explored non-invasive surrogates of RV-PA coupling [13,21]. A recently validated index of RV-PV coupling, TAPSE vs. PAAT, that accounts for the work expended by a force through displacement, was decreased in children with PH [13,14]. This factor correlated with invasive RHC measures of RV performance, pulmonary hemodynamics, and RV-PA coupling [13,14]. We assessed the echocardiographic derived relationship of TAPSE to PAAT in both the adult and neonatal mice models.

### 2.5. Cardiac Catheterization

Adult neonatal mice were anesthetized with Isoflurane (2% maintenance) + pancuronium (1 mg/kg given once). This anesthesia produces a near-physiological heart rate of 500 beats/min, while still allowing for a surgical plane of anesthesia. The mice were intubated and ventilated with a Harvard ventilator set at 200–400 μL. The right jugular vein was identified in the region of the neck and was cannulated with a 1.6 French high fidelity micromanometer pressure-volume catheter (SciSense Advantage System, London, ON, Canada). The catheter was advanced into the right atrium and through the tricuspid valve into the RV to assess pressures. Continuous RV systolic and diastolic pressures were recorded and analyzed with SciSense LabScribe2 version 2.0 analysis software. The cardiac catheterization operators were blinded to the mouse groups.

### 2.6. Cardiac Tissue Processing

Mice subjected to hypoxia were euthanized with a lethal dose of ketamine and xylazine and exsanguinated by cutting the abdominal aorta. The trachea was dissected and cannulated, lungs were fixed via intra-tracheal inflation with 10% phosphate-buffered formalin at a pressure of 20 cm H_2_O for 10 min. Heart and lungs were next immersed in fresh fixative overnight at room temperature. Lung samples were dehydrated in ethanol and xylene, embedded in paraffin, cut in 6 μm sections, and stained with hematoxylin and eosin (H&E).

### 2.7. Immunohistochemistry and Immunofluorescence

Six μm sections were prepared from paraffin-embedded tissues. Sections were deparaffinized and re-hydrated. Antigen retrieval was performed using a pressure cooker and citrate buffer (pH 6.0). Primary antibodies for αSMA (M0851, Dako North America, Carpinteria, CA, USA) was added to blocking buffer and slides were incubated overnight at 4 °C. Colorimetric reaction was performed using DAB staining (Vector Labs, Burlingame, CA, USA) and sections were counterstained with hematoxylin. Primary antibodies for CD31 (dia310, Dianova, Hamburg, Germany) and Cy3 conjugated-αSMA (C6198, Sigma-Aldrich, St Louis, MO, USA) was utilized as described above for immunofluorescence. Immunofluorescent imaging was performed using a Zeiss Apotome and image processing was performed using Zeiss Axioplan software version 3.7.

### 2.8. Whole Slide Scanning

Digital scanning of whole slides was performed using a Nanozoomer 2.0 HT digital slide scanner (Hamamatsu, Bridgewater, NJ, USA), available through the Washington University Hope Center Alafi Neuroimaging Lab (NIH Shared Instrumentation Grant (S10 RR027552)). Images were captured on 40× objectives; high-resolution images were captured and analyzed using NDP.view2 software version 2.8.24.

### 2.9. Measurement of Ventricular Weights

Atria and outflow tracts were removed from the hearts, and the RV was carefully excised following their septal borders. The weight mass of the RV, and the remainder of the heart, left ventricle (LV) + septum (S), were recorded. Atria were trimmed, and RV hypertrophy was measured by weighing the RV relative to the LV + S and the RV or LV + S relative to the animal’s body weight.

### 2.10. Statistics Analysis

All values are presented as mean ± SEM. Comparison between different groups was performed by unpaired Student’s *t*-test. For multiple comparisons, we utilized two-way ANOVA with Bonferroni’s corrections for multiple comparisons using GraphPad Prism software (ver. 7.0; GraphPad, La Jolla, CA, USA). Differences with *p* < 0.05 were considered significant.

## 3. Results

### 3.1. Echocardiographic Assessment of Hypoxia-Induced PH in Adult Mice

**Figure 1:**
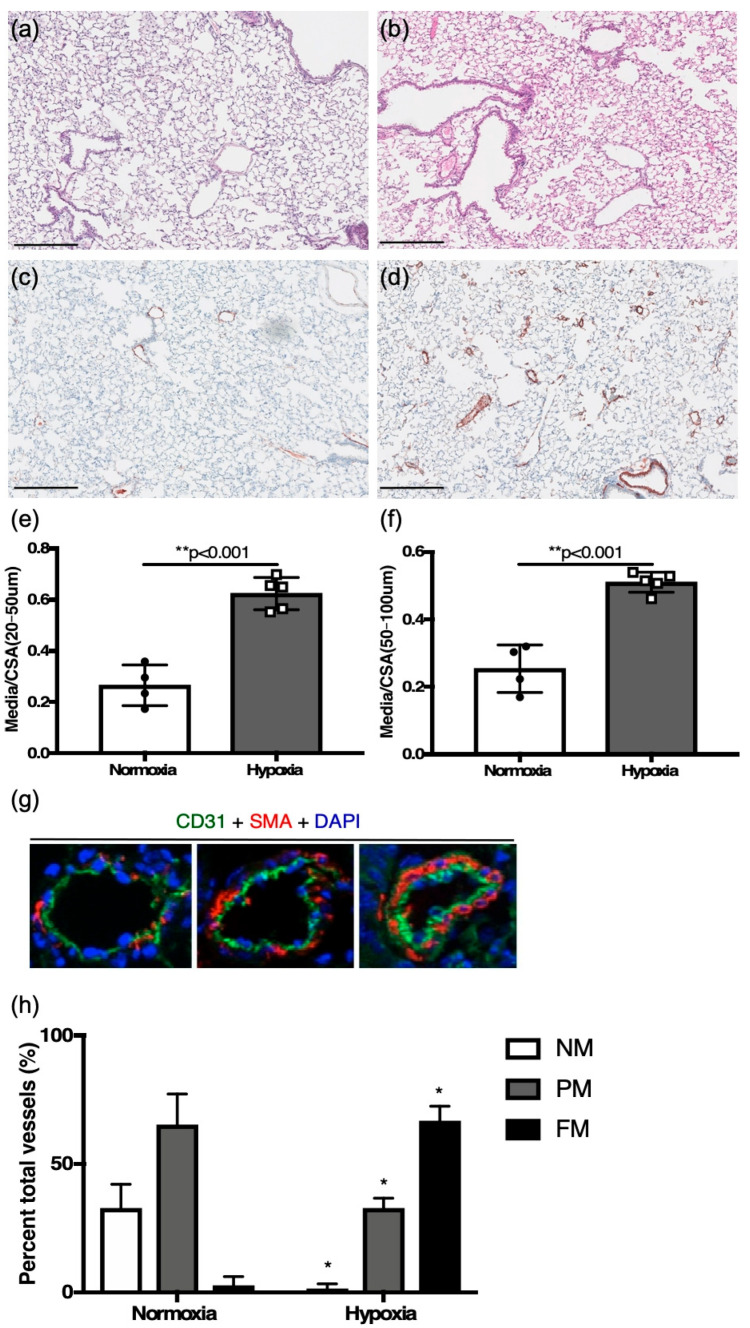
Six-week-old adult mice. (**a**,**c**) changes in lung tissue in normoxia with hematoxylin and eosin, and colorimetric smooth muscle actin immunostaining (brown), respectively; (**b**,**d**) hypoxia challenged mouse lung sections with hematoxylin, and colorimetric smooth muscle actin immunostaining (brown), respectively (scale bar = 25 μm). (**e**) Vessel wall thickening as assessed by medial thickness normalized to vessel cross-sectional area for distal smaller vessels, and (**f**) proximal larger vessels. (**g**) Immunofluorescence staining for CD31 (green), smooth muscle actin (red), DAPI (blue) (scale bar = 5 μm), used for identifying vessels as non-, partial-, or fully muscularized between (**h**) hypoxia challenged mice and controls. * *p* < 0.01, ** *p* < 0.001.

Six-week-old mice that were challenged with two weeks of hypoxia at 10% FiO_2_ manifested significant PH with increased vessel medial wall thickening (Figure 1a–h).

Compared to control littermates in normoxia, medial to total cross-sectional area was increased in distal vessels (20–50 μm) (0.62 ± 0.03, *n* = 4 vs. 0.26 ± 0.04, *n* = 4, *p* < 0.001) and proximal vessels (50–100 μm) (0.51 ± 0.01, *n* = 5 vs. 0.25 ± 0.04, *n* = 4, *p* < 0.01) in hypoxia-challenged mice (Figure 1e,f). The degree of neomuscularization in hypoxic mice, as compared to control littermates in normoxia, also showed significant muscularization of pulmonary vessels (Figure 1g,h).

**Figure 2:**
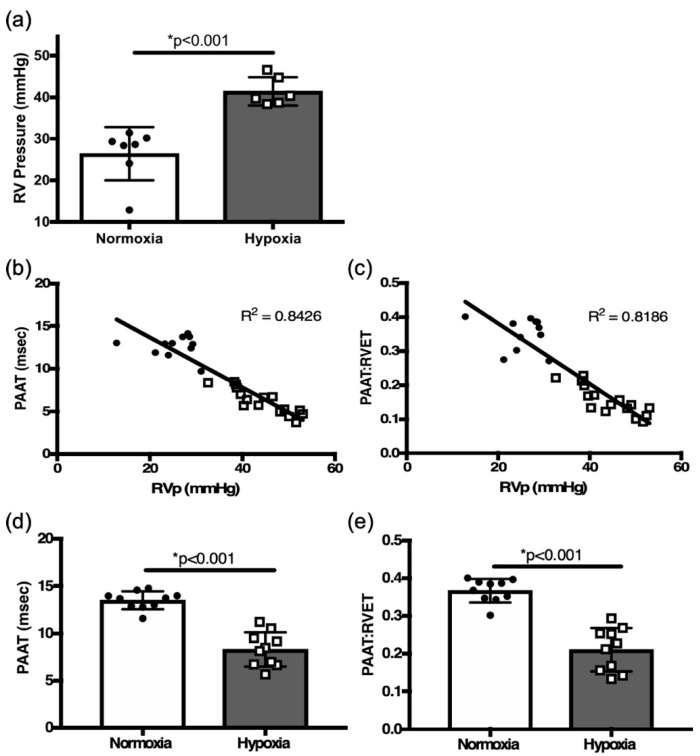
(**a**) Right heart catheterization (RHC)-derived RVSP comparison between normoxia and hypoxia exposed adult mice. Correlation plots between right heart catheterization-derived pulmonary hemodynamics and (**b**) pulmonary artery acceleration time (PAAT), (**c**) PAAT: RV ejection time (PAATi) ratio from all mice (normoxia and hypoxia). Comparison of PAAT (**d**) and PAATi (**e**) in hypoxia challenged mice vs. controls. * *p* < 0.01.

To investigate the development of PH, RVSP, as a surrogate of PASP, was determined by RHC. Two weeks of hypoxia challenge led to a significant increase in RVSP when compared to normoxia controls (41.33 ± 1.396, *n* = 6 vs. 26.34 ± 2.416 mmHg, *n* = 7, *p* < 0.001, Figure 2a). No differences in RVSP were noted between adult male (*n* = 2) and females (*n* = 5). Hypoxia exposure did not affect whole adult animal weight.

Echocardiographic assessment by PAAT of PASP inversely correlated with RVSP measured by RHC in six-week-old mice (R[2] = 0.84, *p* < 0.01, Figure 2b). The PAATi also correlated with RVSP (R[2] = 0.82, *p* < 0.01, Figure 2c). Two weeks of hypoxia exposure led to a significant decrease in PAAT and PAATi when compared to controls in normoxia (8.28 ± 0.57 msec, *n* = 10 vs. 13.5 ± 0.30 msec, *n* = 10, *p* < 0.001, Figure 2d; 0.210 ± 0.018 vs. 0.366 ± 0.010, *n* = 10, msec, *n* = 10, *p* < 0.01, Figure 2e). PAAT and PAATi provide good estimates of RVSP; decreases in their values proved to be reliable indicators of the development of PH in response to hypoxia in adult mice.

### 3.2. Echocardiographic Evaluation of Hypoxia-Induced RV Hypertrophy and RV Function in Adult Mice

**Figure 3:**
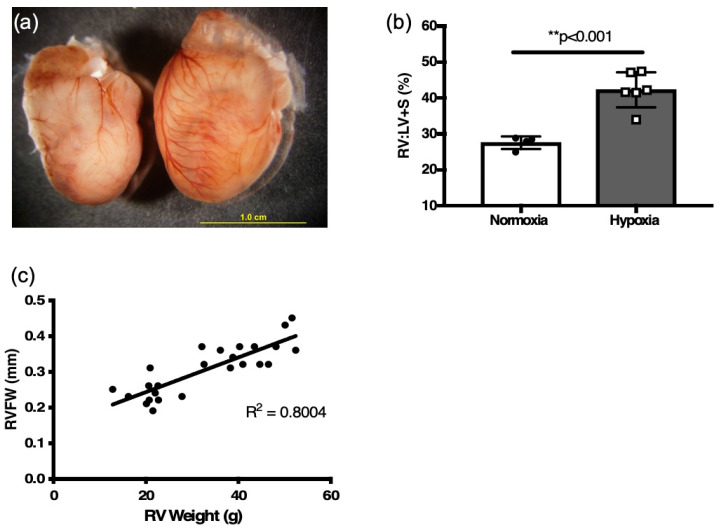
Two weeks hypoxia induces RV hypertrophy in six-week adult mice. (**a**) Representative heart of hypoxia challenged mouse (right) and normoxia control (left). (**b**) weight ratio of right ventricle (RV) to left ventricle plus septum (LV + S) after 2 weeks of hypoxia and age-matched normoxia controls, as an index of RV hypertrophy (*n* = 4–7; ** *p* < 0.001) (**c**) Correlation plot between RVFW thickness and RV weight from all mice (normoxia and hypoxia).

Morphologically, remodeling occurred in the six-week-old mice exposed to two weeks of hypoxia, as RV:LV + S was significantly increased compared to the normoxia mice (*p* < 0.001, Figure 3a,b, Table 1).

**Figure 4:**
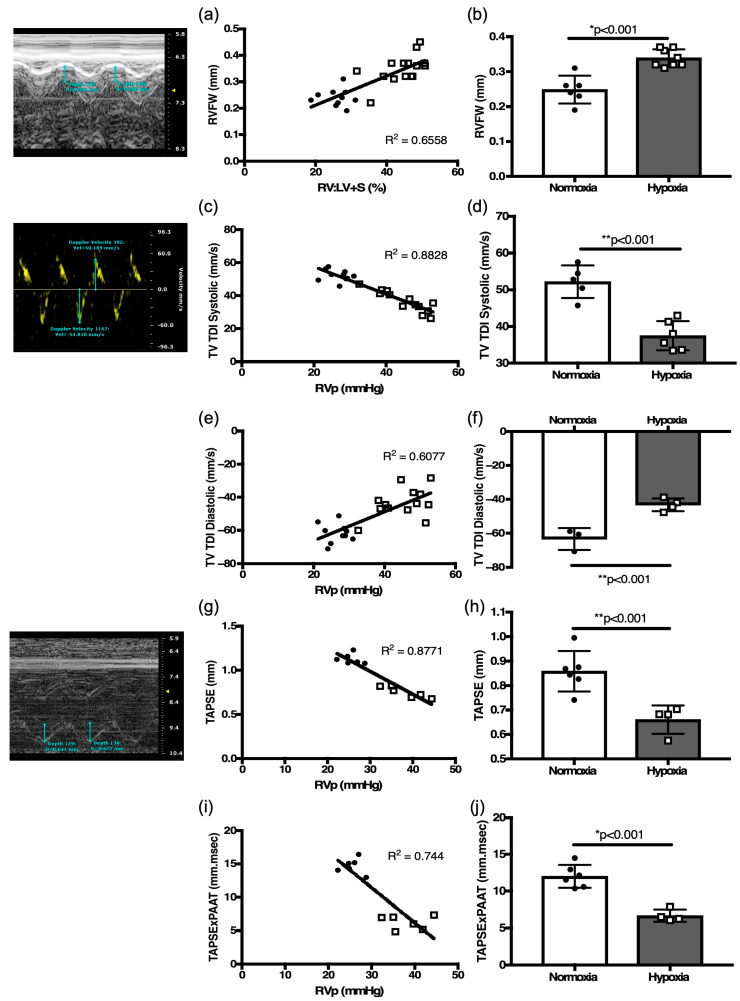
Validation of echocardiography-derived RV function parameters. (**a**) Correlation plot of echocardiography derived RV free wall thickness (RVFW) with weighed RV:LV + S ratio. (**b**) Comparison of RVFW thickness as a surrogate of RV hypertrophy in hypoxia-challenged mice as compared to normoxia controls. Correlation plots between right heart catheterization-derived RV pressure and tricuspid valve tissue Doppler index (TV TDI) in (**c**) systole, (**e**) diastole. (**d**) Comparison of (**d**) systolic TV TDI, and (**f**) diastolic TV TDI in hypoxia-challenged mice as compared to normoxia controls. Correlation plots between right heart catheterization-derived RV pressure with (**g**) Tricuspid Annular Plane Systolic excursion (TAPSE), and with (**i**) TAPSE × PAAT. Comparison of (**h**) TAPSE and (**j**) TAPSE × PAAT as a surrogate of RV capacity for work in hypoxia-challenged mice as compared to normoxia controls. Representative echocardiography images accompany respective measurements for RVFW, TDI, and TAPSE. * *p* < 0.01. ** *p* < 0.001.

There was a positive correlation of RVFW thickness derived by M-mode echocardiography to the RV mass (R[2] = 0.80, Figure 3c) and RV:LV+S ratio (R[2] = 0.66, *p* < 0.01, Figure 4a) obtained by weighing tissue. RVFW thickness was also significantly increased in hypoxia challenged mice compared to normoxia mice (*p* < 0.001, Figure 4b, Table 2).

RV function defined by TV TDI systolic peak velocity inversely correlated with RVSP values (R[2] = 0.88, *p* < 0.01, Figure 4c). TV TDI diastolic velocity correlated with RVSP (R[2] = 0.61, *p* < 0.01, Figure 4e). In response to hypoxia, TV TDI systolic peak velocity was decreased compared to normoxia mice (*p* < 0.001, Figure 4d, Table 2), and the magnitude of TV TDI diastolic velocity was also decreased in the hypoxia exposed mice (*p* < 0.001, Figure 4f, Table 2). TAPSE inversely correlated with RVSP (R[2] = 0.88, *p* < 0.01, Figure 4g). In response to hypoxia, TAPSE was decreased compared to normoxia controls (*p* < 0.001, Figure 4h, Table 2).

The concept of RV contractile reserve is denoted by a change in TAPSE vs. RVSP ratio under stress [21] RV reserve is a function of work (W) and can be estimated by multiplying force (F) and. distance (D), (W = F × D) [13,14]. Since pressure is the force applied per unit area, it is more convenient to use pressure rather than force to describe the influences upon fluid behaviors. PAAT, as a reliable estimate of PASP can be exchanged for force. TAPSE, as a measure of RV function, can be substituted for displacement. Thus, the following equation, TAPSE × PAAT, provides a measure of RV-PA coupling. We demonstrate an inverse correlation of TAPSE × PAAT with RVSP (R[2] = 0.74, *p* < 0.01, Figure 4i). In response to hypoxia, TAPSE × PAAT significantly decreased in hypoxia mice compared to normoxia controls (*p* < 0.01, Figure 4j, Table 2), suggesting a significant decrease in RV work capacity secondary to hypoxia induced PH. These echocardiographic indices in adult mice proved to be consistent indicators of changes in RV morphology and function.

### 3.3. Neonatal Hypoxia Results in Pulmonary Hypertension

**Figure 5:**
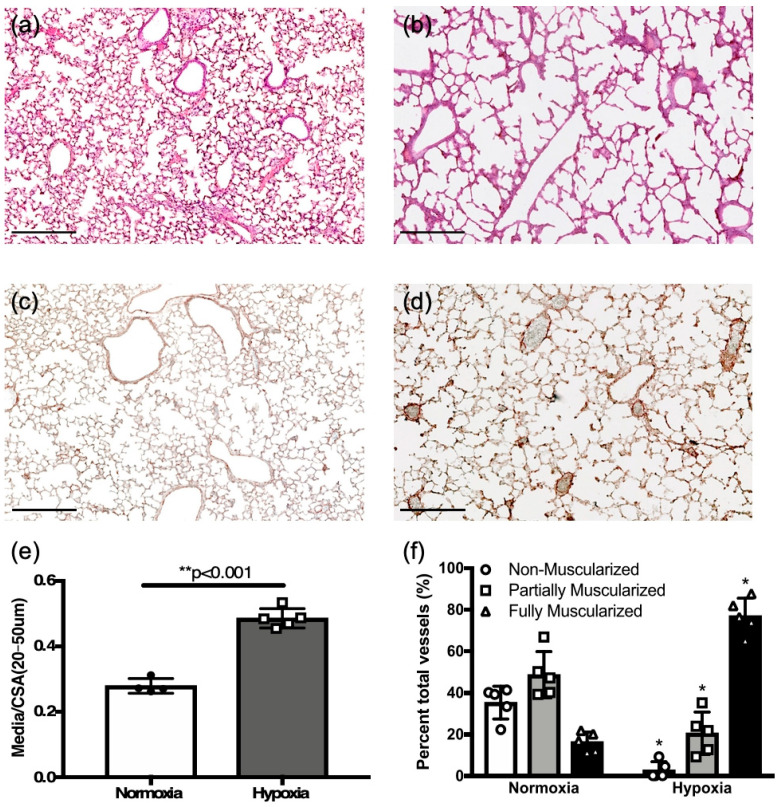
Chronic hypoxia induces pulmonary hypertension changes in neonatal mice. (**a**,**c**) Representative normoxia and (**b**,**d**) hypoxia challenged neonatal mouse lung sections with hematoxylin and eosin, and colorimetric smooth muscle actin immunostaining (brown), respectively (scale bar = 25 μm). (**e**) Comparison of vessel wall thickening as assessed by medial thickness normalized to vessel cross-sectional area in hypoxia challenged neonates as compared to normoxia controls. (**f**) Comparison of percent vessels that are non-, partial-, or fully muscularized between hypoxia challenged mice and controls. * *p* < 0.05; ** *p* < 0.001.

To determine whether hypoxia exposure induces PH changes in neonatal mice, newborn mice (P0), together with the dam, were placed in 10% FiO_2_ for two weeks. Histological analysis of hypoxia-exposed mice showed alveolar simplification (Figure 5a,b) and increased vessel medial wall thickening (Figure 5c,d). 

Medial to total cross-sectional area was increased in hypoxia challenged mice compared to control newborns in normoxia (0.48 ± 0.01, *n* = 5 vs. 0.28 ± 0.01, *n* = 4, *p* < 0.01, Figure 5e). Hypoxia caused a significant shift towards fully muscularized vessels (Figure 5f), as was seen in adult mice exposed to hypoxia (Figure 1h).

### 3.4. Hypoxia Induces PH in Neonatal Mice

**Figure 6:**
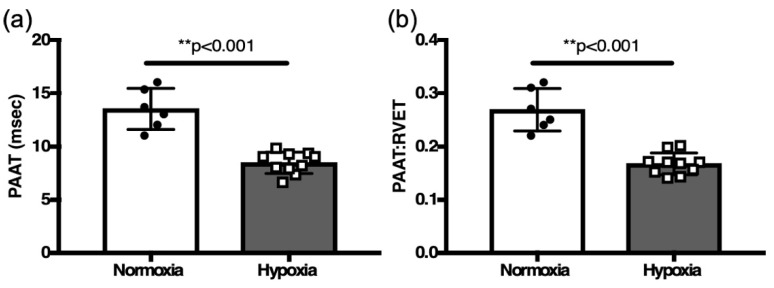
Comparison of (**a**) PAAT and (**b**) PAAT:RVET in hypoxia challenged neonates vs. normoxia controls. ** *p* < 0.001.

Since RHC is not technically feasible in 2-week-old mice, we used PAAT indices that were validated as reliable surrogates PASP in adult mice. In response to two weeks of hypoxia, the PAAT and PAATi were both decreased in hypoxia challenged neonatal mice compared to controls in normoxia (8.4 ± 0.32 msec, *n*=10 vs. 13.5 ± 0.78 msec, *n* = 6, *p* < 0.001) and (0.17 ± 0.01, *n* = 10 vs. 0.27 ± 0.02, *n* = 6, *p* < 0.001, Figure 6).

### 3.5. Neonatal Hypoxia Causes RV Hypertrophy and Diminished RV Function

**Figure 7:**
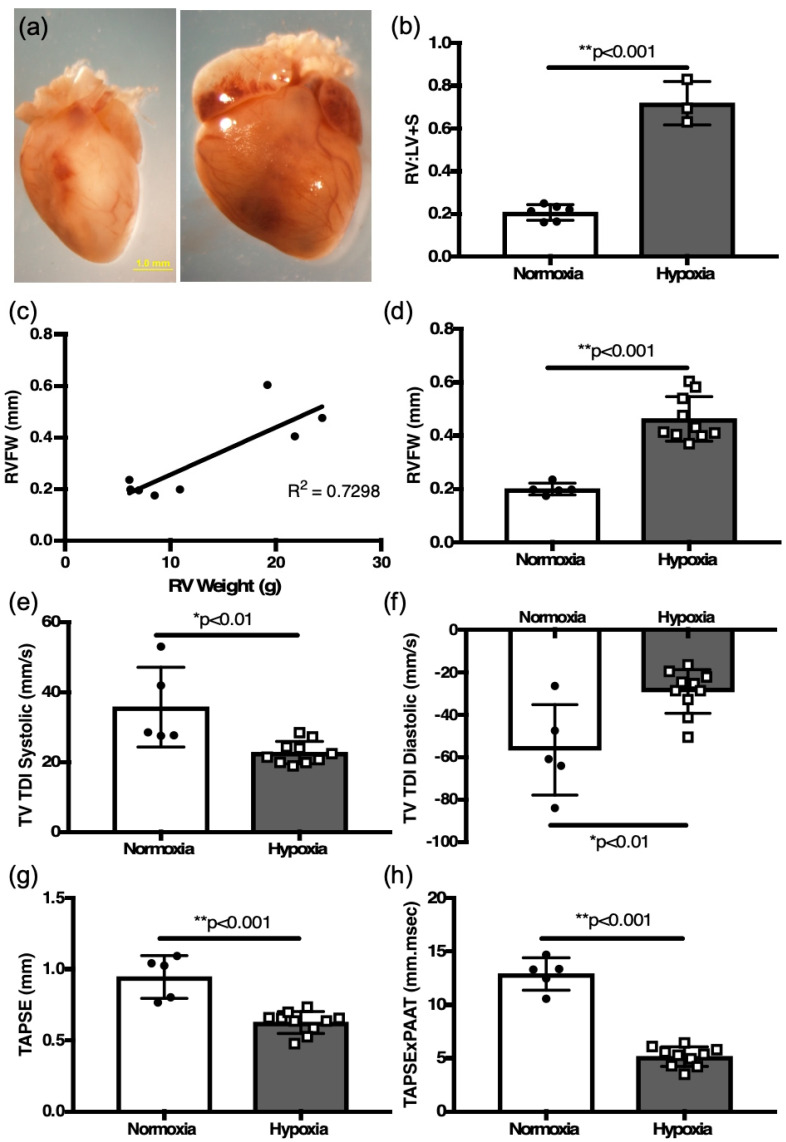
Two weeks hypoxia induces RV hypertrophy and RV function changes in neonatal mice. (**a**) Representative heart of hypoxia challenged neonatal mouse pup (right) and normoxia control (left). (**b**) weight ratio of right ventricle (RV) to left ventricle plus septum (LV + S) after 2 weeks of hypoxia and age-matched normoxia controls, as an index of RV hypertrophy. (**c**) Correlation plot between RVFW thickness and RV weight from all mice (normoxia and hypoxia). Comparisons of (**d**) RVFW thickness, (**e**) systolic TV TDI, (**f**) diastolic TV TDI, (**g**) TAPSE, and (**h**) TAPSE × PAAT as a surrogate of RV capacity for work in hypoxia-challenged mice as compared to normoxia controls. * *p* < 0.01, ** *p* < 0.001.

Similarly to adult mice, remodeling, manifested by marked cardiomegaly, occurred in neonatal mice following two weeks of hypoxia exposure in neonatal mice (Figure 7a).

Measurement of the RV:LV + S weight ratio was increased in the hypoxia-exposed mice compared to the controls (0.72 ± 0.06 g, *n* = 3 vs. 0.21 ± 0.01 g, *n* = 6, *p* < 0.01, Figure 7b). Hypoxia exposure decreased whole animal weight (normoxia 5.32 ± 0.06 g, *n* = 6 vs. hypoxia 4.82 ± 0.14 g, *n* = 10, *p* < 0.01), and increased RV mass (normoxia 7.65 ± 0.6 g, *n* = 6 vs. hypoxia 21.8 ± 1.0 g, *n* = 3, *p* < 0.003). There was no difference in LV+S mass (normoxia 37.4 ± 2.2 g, *n* = 6 vs. hypoxia 30.6 ± 1.8 g, *n* = 3). There was a positive correlation of RVFW thickness derived by M-mode echocardiography to the RV mass (R[2] = 0.73, Figure 7c). Echocardiographic measurement of RV free wall thickness was also increased in hypoxia-challenged newborn mice compared to control newborns in normoxia (0.46 ± 0.03 mm, *n* = 10 vs. 0.20 ± 0.01 mm, *n* = 5, *p* < 0.01) (Figure 7d).

Measurements of RV function, TV TDI in systole (22.67 ± 1.0 mm/s, *n* = 10 vs. 35.7 ± 5.1 mm/s, *n* = 5, *p* < 0.01, Figure 7e), TV TDI in diastole (−29.2 ± 3.3 mm/s, *n* = 10 vs. −56.7 ± 9.5 mm/s, *n* = 5, *p* < 0.01, Figure 7f), and TAPSE (0.944 ± 0.06 mm, *n* = 5 vs. 0.624 ± 0.06 mm, *n* = 10, *p* < 0.001, Figure 7g), were all decreased in the hypoxic neonatal mice. Finally, the TAPSE × PAAT relationship was also significantly decreased in the hypoxic neonatal mice (5.13 ± 0.29 mm/s, *n* = 10 vs. 12.85 ± 0.68 mm/s, *n* = 5, *p* < 0.001, Figure 7h). There were no differences in RV hypertrophy or echocardiographic measures of RV function noted between neonatal male (*n* = 10) and females (*n* = 5).

## 4. Discussion

In this study we demonstrated from simultaneous RHC and echocardiography in an adult mouse model of PH that (1) PAAT is a reliable estimate of RVSP to characterize changes on RV afterload, (2) M-mode measured RVFW thickness is a sensitive marker of RV hypertrophy, confirmed by histopathology, and (3) TAPSE and PAAT derived factors are markers of appreciable changes in RV functional reserve. When applied to hypoxia-exposed neonatal mice, these echocardiographic indices provided sensitive diagnostic markers of hypoxia induced PH and changes in RV morphology and function. Well-validated, non-invasive echocardiographic measures of RV performance can serve as endpoints for studying the progression of PH in animal models and evaluating the effectiveness of various treatment strategies through preclinical therapeutic modeling.

### 4.1. Echocardiographic Assessment of Hypoxia-Induced PH, RV Hypertrophy, and RV Dysfunction in Adult Mice

RV remodeling and functional changes in response to the development of PH are the most important determinants of prognosis in patients with PH [22,23]. Maladaptive remodeling of the RV in response to increasing PVR is a common phenotype of PH. Cardiomegaly secondary to RV hypertrophic remodeling initially maintains cardiac output; however, due to disproportionate remodeling relative to increasing PVR, RV function eventually declines leading to right heart failure. Early detection of PH by screening with assessment of RV afterload, function, and remodeling provide an opportunity for prompt intervention. However, diagnosis of PH in premature neonates and mice is difficult due to a lack of reliable non-invasive measures that precisely characterize pulmonary hemodynamics and RV performance. Cardiac catheterization is the standard reference modality, but in premature neonates it is an invasive procedure with risk for significant morbidity, and in mice it is a terminal procedure with a minimum weight requirement (18 g) for its feasibility. Although echocardiographic assessments of RV structure and function has become a routine in the clinical evaluation and follow-up of patients with PH, the validity of many echocardiographic indices for such assessment compared to the standard reference of RHC have not been evaluated in mice [24].

To our knowledge, this is one of the first studies of hypoxia-induced PH in mice that simultaneously compares RHC and echocardiography indices of pulmonary hemodynamics and RV performance, in addition to confirming it against histopathological evidence of PH and RV remodeling. In this study, we demonstrated that echocardiographic surrogates of PASP, PAAT and PAATi, provide a reliable estimate of RHC derived RVSP. PAAT measures have been utilized to assess PH in mouse models [9,17,24,25]. Our group has also previously validated PAAT and PAATi as reliable estimates of pulmonary hemodynamics with simultaneous RHC in neonates and children with PH [2]. Echocardiographic derived RVFW thickness, an index of RV remodeling, closely correlated with characteristic histological phenotypes of PH in mice: RV:LV + S mass ratios, increased medial to cross-sectional area of vessels, and neo-muscularization of vessels. Our study extends the knowledge that RV wall thickness represents a sensitive index of RV remodeling and increasing RV mass [24].

We further showed that RV functional changes in response to the development of PH can be assessed by TAPSE, TV TDI systolic and diastolic velocities for early detection of maladaptive changes in RV performance. Myocardial velocities of TV annulus by TDI have been validated as a reliable tool to assess RV function in neonates with PH [6]. Richardson et al. demonstrated that TV TDI peak systolic and early diastolic velocities reliably predict PH in infants [6]. The data presented in this study suggests that TV TDI systolic and diastolic velocities are indicators of RV dysfunction secondary to hypoxia induced PH in mice and provide a tool for early detection of maladaptive changes in the RV in response to the development of PH in mice. In adults with PH, RV TDI systolic velocity has been shown to correlate with RV systolic function assessed by TAPSE [26]. In addition, temporal changes in the TAPSE values correlate with early PH in premature infants with BPD [27], and is a significant predictor of outcome in adult patients with pulmonary arterial hypertension [26] or cardiomyopathy associated heart failure [28]. Our study demonstrates that TAPSE can be reliably obtained in mice, despite the technical challenges of visualization of the RV that stem from the shape of the chest and the anatomic orientation of the RV within the thoracic cavity in mice. When obtained correctly, TAPSE is a reliable indicator of functional changes in RV secondary to PH.

### 4.2. Echocardiographic Assessment of Hypoxia-Induced PH, RV Hypertrophy, and RV Function in Neonatal Mice

We applied the echocardiographic measures of pulmonary hemodynamics and RV performance validated against RHC, and confirmed with histopathology, from the adult arm of the study to a neonatal PH mouse model. This is only the second study to utilize echocardiography to characterize PH in neonatal mice [24]. All of the previous work in the neonatal mouse model of PH relies on histology or a terminal thoracotomy procedure with direct percutaneous catheterization to confirm the diagnosis. A summary of the studies that have assessed RV function or morphology in a neonatal mouse lung injury model are presented in Table 2 [6,24,29,30,31,32,33,34,35,36]. In our study, following two weeks of hypoxia at 10% FiO_2_ in neonatal mice, we found that PAAT and PAATi were decreased, suggesting increases in PVR and RV afterload. We showed that PAAT ranges are similar between adult and neonatal mice, both at baseline and when challenged with hypoxia. We also demonstrated that the magnitudes of TAPSE and PAAT product were reduced, indicating decreased RV functional reserve. RV free wall thickness was increased, reflecting the expected RV hypertrophy observed with the adult PH mouse model. These indices also correlated with the histological diagnosis of PH and RV morphology in neonatal mice.

**Table 2:** Brief summary of studies on neonatal lung injury models and methods of assessment for pulmonary hypertension.

Author (year)	Age	Lung Disease Model	Mechanism	Adult Mice Comparison	PH Assessment
Yang (2015) [6]	p0–p14	11%, 2 weeks	IGF-1	No	Thoracotomy with direct RV catheterization, Histology
Reynolds (2016) [24]	p0–p14	70%, 2 weeks	BPD	No	Echocardiography Histology
Ambalavanan (2005) [29]	p0–p14	14%, 2 weeks	ETAR	No	Histology *
Young (2009) [30]	p0–p14	12%, 2 weeks	CXCR4	No	Thoracotomy with direct RV catheterization Histology
Bierer (2011) [31]	p2–p14	Hyobaric	NFATc3	Yes	Histology
Sartina (2012) [32]	p0–p14	12%, 2 weeks	CXCR7	No	Thoracotomy with direct RV catheterization Histology
Gupta (2015) [33]	p0	Hyperoxia	SOD2	No	Histology
Sun (2016) [34]	p0–p14 + 4 weeks	11%, 2 weeks	IGF-1	Yes	RV catheterization via IJ at 6 weeks Histology
Young (2016) [35]	p0–p14 + 4 weeks	12%, 2 weeks	SCF	No	Thoracotomy with direct RV catheterization Histology
Sherlock (2018) [36]	p2–p22	Bleomycin	SOD	No	Histology
Woo (current study)	p0–p14	10%, 2 weeks	BPD	Yes	Echocardiography Catheterization (adults only), Histology

* Histology, Fulton’s index. RV, right ventricle; IJ, internal jugular; ETAR, endothelin-A receptor; CXCR4, chemokine receptor type 4; CXCR7, chemokine receptor type 4; SCF, stem cell factor; IGF-1, insulin growth factor; SOD, superoxide dismutase; NFATc3, nuclear factor of activated T cells isoform c3.

There was an exaggerated response of the RV to hypoxia in neonatal mice compared to the adult mice. Compared to children and adults, the neonatal myocardium is characterized by systolic dysfunction due to an immature and inefficient contractile apparatus and diastolic dysfunction due to the lack of elastic compliant tissue and a preponderance of stiff fibers. The response of the RV to postnatal stressors, e.g., changes in loading conditions and contractility, is further exacerbated in the premature infants. Since the neonatal RV is more sensitive to changes in loading conditions than the adult RV, and with the observed exaggerated response of the RV to hypoxia in neonatal mice, as compared to adult mice, we propose that the RV may be independently responding to hypoxia, rather than playing an indirect role in the PH [37,38].

### 4.3. RV-PA Coupling Mice

This is the first study of hypoxia induced PH in mice to characterize the relationship between the RV and its afterload with a novel validated measures RV-PA coupling. Coupling of the RV-PA axis is predicated on the work-energy principle that scientifically implies that a transfer of energy between two related structures is equal to the work expended by a force through displacement [37,39,40]. Since the RV is a pulsatile pump, its efficiency or work depends on proper hemodynamic coupling to the compliant pulmonary arterial circulation [39,40]. Our group recently demonstrated that the relationship of TAPSE to PAAT, as an index of the length-force relationship, correlated with invasive RHC measures of RV performance, pulmonary hemodynamics and RV-PA coupling [13,14]. In this study we demonstrated that both the adult and neonatal mice exposed to two weeks of hypoxia have decreased TAPSE × PAAT factors when compared to normoxia controls. The advantage of a factor that relates TAPSE to PAAT is that “examining the individual components of the RV-PA coupling index provides a comprehensive evaluation of whether alterations are caused by arterial properties, ventricular properties, or both.” [13,14]. The TAPSE × PAAT relationship integrates contractility with all the determinants of RV afterload (resistance, compliance, and impedance). A characterization of the RV-PA axis with length–force relationship will allow RV-PA coupling changes to be measured serially for coupling trajectory; this may identify the etiology and potentially expand the scope of conditions in which coupling can be investigated in mice and neonates.

### 4.4. Clinical Implications

The findings may have important clinical implications in preclinical and clinical studies and are particularly relevant for clinical management and research in pediatric cardio-pulmonary diseases. While there is a growing recognition of the clinical importance of hypoxic lung disease in neonates, there is a paucity of robust non-invasive methods to characterize the cardio-pulmonary phenotypes in neonatal and mouse PH. This notion is further supported by the recognition that histologic studies to determine disease are either terminal (mice), invasive (neonates) and require large number of subjects for longitudinal studies. In addition, the small size of newborn infants and neonatal mice preclude the use of invasive hemodynamic measurements. We feel that those non-invasive indices of pulmonary hemodynamic, RV structural and RV functional assessment that were validated in this study can be applicable for clinical assessment RV phenotype in neonatal mice, premature infants, and children with PH. Notably, the finding that hypoxia alone significantly impaired RV function—evidenced by reduced TAPSE and systolic TDI—has important clinical implications for assessing RV dysfunction in patients with respiratory distress, even in the absence of pulmonary hypertension.

Furthermore, neonatal mouse pups provide several benefits as a model for hypoxia-induced PH or hyperoxia-induced bronchopulmonary dysplasia. Mouse lung development from birth to 2 weeks of age is similar to lung development from week 24 of gestation through 2 years of age [41,42]. Thus, to characterize neonatal chronic hypoxia mouse model adequately mimics pulmonary vascular development and the maladaptive response to hypoxia in infants. There are several additional pre-clinical and clinical models for which to apply these measures. One such example is that neonatal hearts are capable of recovering from ischemic insults up to seven days of age [43]. Experiments are currently underway to evaluate the capacity of the RV to recover if the neonatal mice are removed from hypoxia at the end of two weeks. Experiments are planned to assess whether RV remodeling occurs through hypertrophy or myocyte hyperplasia or both, and to evaluate the cardiac fibrosis responses of adult and neonatal mice to hypoxia.

### 4.5. Limitations

There are several important limitations to consider in this study. There is no consensus on which hypoxia or hyperoxia mouse model most closely mimics the PVD and PH observed in premature infants. In this study we employed a common model of chronic hypoxia following two weeks of hypoxia in 10% FiO_2_ to mimic what would be seen in premature infants with severe BPD. We recognize that other studies have utilized one, three, and four week period of hypoxia [6,25,44,45], with several others exploring models of hyperoxia induced chronic lung disease and PH [24]. We recognize that alveolar and vascular development is a heterogeneous process, and our study only made assessments at single time point during lung development. By only examining neonatal mice at one time point (p14), it is likely that we are missing sensitive abnormalities in alveolarization that may have either resolved or have yet to develop with “compensatory alveolar growth” [36]. This diverse phenotypic manifestation is similar to the process that is seen with some preterm infants with BPD, who can present with no apparent lung disease or severe pulmonary hypertension. Therefore, the echocardiographic parameters tested in this study might only be applicable to this specific model of hypoxia and would likely need to be validated for different durations of hypoxia and hyperoxia exposure. Although TAPSE and TDI are well-established measures of RV function, additional parameters—such as fractional area change and strain-based deformation analysis—may offer further insights into RV performance. Future work is needed to provide correlation analyses between RV strain parameters, the severity of hypoxia, and the extent of RV fibrosis to substantiate the proposed pathophysiological mechanisms. Although we recognize that sex differences play a role in neonatal lung injury, our study was underpowered to test this question. The role of maternal hypoxia on nursing pups is an area of ongoing research; our group is currently investigating protocols in which dams are alternated between normoxia and hypoxia. Finally, the effects of intermittent periods of hypoxia and chronic sustained hypoxia and on lung alveolar development are also not know at this stage, but appear to lead to alveolar simplification [46]. Future studies are required to explore the role of intermittent hypoxia protocols and their effects on neonatal mice.

## 5. Conclusions

The echocardiographic indices of RV hemodynamics, morphology, and function provide reliable estimates of invasive RVSP and RV phenotype in hypoxia-induced PH in adult mice. When applied to hypoxia-exposed neonatal mice, these measures robustly correlated with histological evidence of PH and RV remodeling. These noninvasive indices may permit the characterization of the evolution of PH in genetically modified mice.

## Figures and Tables

**Table 1:** Measurements and echocardiographic values of hypoxia-induced RV hypertrophy and RV function in adult mice.

	Normoxia	Hypoxia
RV ± SEM, g	21.4 ± 0.3 (*n* = 4)	34.5 ± 2.7 (*n* = 6)
LV + S ± SEM, g	78.1 ± 2.3 (*n* = 4)	81.2 ± 4.2 (*n* = 6)
RV:LV + S ± SEM, %	27.5 ± 0.9 (*n* = 4)	42.2 ± 2.0 (*n* = 6)
RVFW thickness, mm	0.25 ± 0.02 (*n* = 6)	0.34 ± 0.01 (*n* = 8)
TV TDI systolic peak velocity, mm/s	52.2 ± 2.0 (*n* = 5)	37.4 ± 1.6 (*n* = 6)
TV TDI diastolic peak velocity, mm/s	−43.3 ± 1.9 (*n* = 4)	−63.4 ± 3.7 (*n* = 3)
TAPSE, mm	0.86 ± 0.03 (*n* = 6)	0.66 ± 0.03 (*n* = 4)
TAPSE × PAAT, mms	11.98 ± 0.63 (*n* = 6)	6.65 ± 0.41 (*n* = 4)

## Data Availability

All data have been presented in this study. Further enquiries can be directed to the corresponding author.

## Abbreviations

The following abbreviations are used in this manuscript:

BPD	Bronchopulmonary dysplasia
PAAT	Pulmonary artery acceleration time
PAATi	Indexed Pulmonary artery acceleration time (PAAT was adjusted to RVET)
PH	Pulmonary hypertension
PVD	Pulmonary vascular disease
RHC	Right heart catheterization
RV	Right ventricle
RV	Right ventricle ejection time
RVSP	Right ventricular systolic pressure
RVFW	Right ventricle free wall
TAPSE	Tricuspid annular plane excursion
TDI	Tissue Doppler imaging
TV	Tricuspid valve
